# Development and Performance Evaluation of an Asphalt Regenerant Derived from Waste Engine Oil Residue

**DOI:** 10.3390/ma16196488

**Published:** 2023-09-29

**Authors:** Rukai Li, Zhansheng Pang, Tianqing Ling, Tengfei Wang

**Affiliations:** 1 School of Civil Engineering, Chongqing Jiaotong University, Chongqing 400074, China; lirukai89@163.com (R.L.);; 2 MOE Key Laboratory of High-Speed Railway Engineering, Southwest Jiaotong University, Chengdu 610031, China

**Keywords:** pavement engineering, waste engine oil residue, asphalt regenerant, road performance

## Abstract

This study assessed the fundamental physical properties and chemical composition of three specific waste engine oil residue (WEORs) asphalt regenerants. Through dynamic shear rheometer and rolling thin-film oven tests, the performance of aged asphalt was evaluated using three key indicators. Thin-layer chromatography investigations probed the WEOR-induced changes in the aging asphalt components, leading to the creation of two novel asphalt regenerants, WEOR-H and WEOR-G. WEOR-G was developed from WEOR-1, liquid rubber, ultraviolet absorber, light shielding agent, and antioxidant, while WEOR-H was formulated from WEOR-2, aromatic oil, and liquid rubber. The study employed differential scanning calorimetry and conventional laboratory tests to analyze the road performance attributes of Ingevity J type regenerant (J), WEOR-G, and WEOR-H. The results indicated that WEORs increase the saturate and aromatic content in asphalt and partially replenish the missing lightweight components of aged asphalt, moderately improving the three key indicators, though the regenerative effect is restricted. Achieving a full restoration of component proportions within aged asphalt to their initial levels proved unattainable, and direct application of any of the three WEORs as asphalt regenerants is impractical. WEOR-H and WEOR-G demonstrated potential in enhancing aged asphalt binder road performance, outpacing three other WEORs. At a 14% dosage, WEOR-G and WEOR-H could increase the 10 °C ductility to 23.5 and 21.4 cm, respectively, effectively counterbalancing the insufficient ability of WEOR-1 and WEOR-2 to restore the low-temperature performance of aged asphalt. Among the regenerants, WEOR-G, possessing superior regenerative effects, the lowest glass transition temperature, and optimal low-temperature deformation resistance, emerged as the most efficacious. This inquiry furnishes vital data support for future applications of WEOR-G asphalt regenerant.

## 1. Introduction

As car numbers rise in China, engine oil usage follows suit. Impurity content in this oil escalates during use, interacting with atmospheric oxygen to generate macromolecular oxides, such as resin and asphaltene, collectively termed ‘waste engine oil’. Developed countries, such as the United States, prioritize waste engine oil regeneration and utilization. Processes, such as centrifugal separation, molecular distillation, flocculation, and membrane treatment, extract light components from waste engine oil, repurposing them for lower-grade engine or lubricating oils. This regenerates and utilizes 75–85% of waste engine oil. However, the remaining 15–25% of heavy components resist effective recycling due to impurity inclusion, forming ‘waste engine oil residue’ (WEOR) [1].

Given the significant consumption of construction materials in road projects, the field of road construction has emerged as a pivotal domain for the resourceful reuse of waste. For instance, waste plastic [2,3], waste rubber [4,5], waste glass [6,7], waste catalysts [8,9], waste cooking oil [10], and so on. International research demonstrates that WEORs can soften aged asphalt and, with specialized processing, enhance aged asphalt performance [11,12,13]. So, the WEORs can be used for asphalt rejuvenation. Qurashi [14] reported that adding WEORs improves road performance of aged asphalt. Ren [15] assessed the rejuvenating effect of waste edible oil and waste engine oil on aged asphalt performance, with results indicating that both waste oils increase adhesion and crack resistance. Moreover, incorporating styrene butadiene rubber can enhance temperature sensitivity. Evaluations by Tabakovic [16] and Li [17] showed that a WEOR can reduce allowable strain and low-temperature limit grading of aged asphalt, and modification technologies have been suggested to augment the regenerative effect of WEORs on aged asphalts. 

Experiments conducted by John D’Angelo [18,19], Wielinski [20,21], Kemas [22], Zamhari [23], Oliveira [24], and Deden [25] have studied the regeneration effect of WEOR on both asphalt and asphalt mixtures. Their results affirm that WEOR plays a significant role in improving the performance of asphalt, particularly in the low-temperature range. Research by Villanueva [26], Zaumanis [23], and Li [27] further extends this understanding, demonstrating various impacts of WEOR, such as an enhanced permeability index, increased creep flexibility and fracture energy, as well as superior low-temperature cracking resistance.

Furthermore, the studies by Xue [28] and Khan [29] have introduced additional aspects, including the use of lignin and the integration of WEOR with other materials. They have revealed insights into the alterations of elasticity and stiffness in asphalt binders and demonstrated the feasibility of designing specific asphalt mixes with up to 100% recycled asphalt pavement (RAP) in combination with WEOR and CRM, meeting standard criteria. These findings not only contribute to the ongoing innovation in waste engine oil utilization, but also underscore the potential for significant improvements in road construction and maintenance.

At present, global scholars have focused their research on three principal indicators of WEOR-modified asphalts, assessing the mixed road performances and confirming the feasibility of employing WEOR as an asphalt modifier. Despite these advancements, investigation into WEOR asphalt regenerant products within China is limited, and challenges related to the transformation of WEORs for road use continue to persist. Furthermore, a previous study proved that the inclusion of waste engine oil can lead to a decreased rutting resistance of the asphalt binder [30]. From this point of view, it is not wise to only use WEOR as an asphalt regenerant. It is necessary to study the introduction of other additives to obtain the asphalt regenerant based upon WEOR.

This study embarks on an examination of three distinct WEORs, produced through two separate processes. Initially, the research scrutinizes the impact of the three varying WEORs on the performance of aging asphalt, followed by the preparation of WEOR-G and WEOR-H asphalt regenerants utilizing two technological pathways to augment the regenerative effect of WEORs on aged asphalt. Finally, the study conducts a comparative analysis of the road performances of regenerant J, WEOR-G, and WEOR-H, with the objective of formulating a strategy to recycle WEORs and bolster waste engine oil utilization. The results show that the asphalt binder regenerated by WEOR-G, composed of WEOR-1, liquid rubber, ultraviolet absorber, light shielding agent, and antioxidant, exhibited the best high- and low-temperature performance. This inquiry furnishes vital data support for the practical implementation of high-performance WEOR regenerative RAP. 

**Figure 1:**
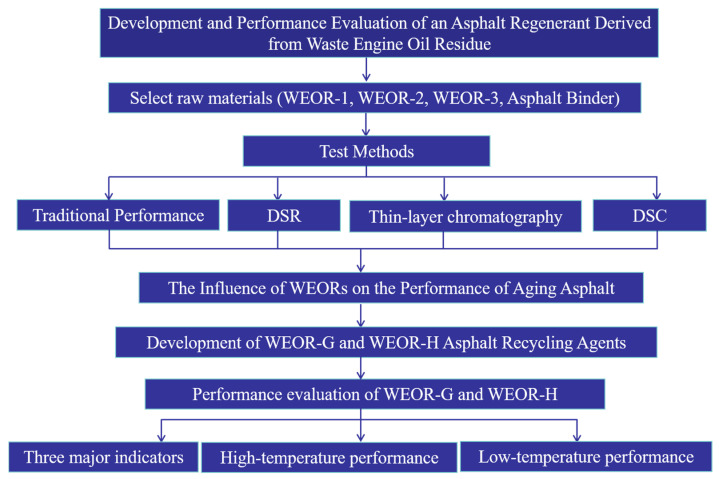
Flowchart of proposed asphalt regenerant development and performance assessment.

The flowchart of this work is shown in Figure 1.

## 2. Experimental

### 2.1. Materials

#### 2.1.1. Waste Engine Oil Residue 

Table 1 presents the essential physical indicators for three classifications of WEORs, designated as WEOR-1, WEOR-2, and WEOR-3. WEOR-1 was derived utilizing the Hopper permeation membrane process, while WEOR-2 was prepared through the molecular distillation technique. Conversely, WEOR-3 was produced using the molecular distillation process. In comparison to the membrane treatment method, the molecular distillation process provides a more thorough separation of waste oil components, resulting in a lower lightweight constituent content and superior economic efficiency.

#### 2.1.2. Asphalt Binder

Li’s study [31] posits that a brief 270-min rolling thin-film oven aging test might correspond to an extended pressure aging vessel evaluation. Employing this approach, aging experiments were carried out on Shell 70# base asphalt. Table 2 delineates the three principal indicators both before and after the asphalt aging. Upon completing the preparation of aged asphalt, varying proportions of WEOR-1, WEOR-2, and WEOR-3 were incorporated to formulate asphalt samples. Subsequently, the impact of WEORs on the road performance of aged asphalt was scrutinized through experimental analysis.

### 2.2. Test Methods

#### 2.2.1. Traditional Performance Evaluations Tests

Traditional performance evaluation methods, such as penetration, softening point, and ductility testing, comply with the ‘Test Specification for Asphalt and Asphalt Mixtures in Highway Engineering’ (JTG E20-2011). Three parallel experiments were performed for each test.

#### 2.2.2. Dynamic Shear Rheometer Tests

The dynamic shear rheometer (DSR) examination utilized the AR2000ex Advanced Rheometer (Waters Technology Co., Ltd., Milford, MA, USA) to measure the complex modulus and phase angle of asphalt samples at temperatures of 52 °C, 58 °C, 64 °C, and 70 °C, thereby determining the rutting indicator. The phase angle characterizes the viscoelasticity of asphalt, with a 0° phase angle indicating fully elastic materials and 90° corresponding to purely viscous fluid materials. The complex modulus is proportional to the asphalt’s high-temperature performance level. A higher level signifies an enhanced ability to resist high-temperature deformation.

#### 2.2.3. Thin-Layer Chromatography Tests

Thin-layer chromatography, in conjunction with a hydrogen flame ion detector [32], executed the four-component analysis on asphalt binders.

#### 2.2.4. Differential Scanning Calorimetry Tests

The differential scanning calorimetry (DSC) test utilized a DSC214 System—Instrument (Netzsch Company, Selb, Germany) to identify the glass transition temperature and phase transformation process. The testing conditions included a sample mass of 10 mg, a temperature range of −60 °C to 150 °C, a heating rate of 10 °C/min, and a nitrogen flow rate of 20 mol/min. The information presented in this article reflects the mean values derived from three parallel test results.

## 3. Analysis of the Influence of WEOR on the Performance of Aging Asphalt

### 3.1. Test Results for Three Major Indicators

**Figure 2:**
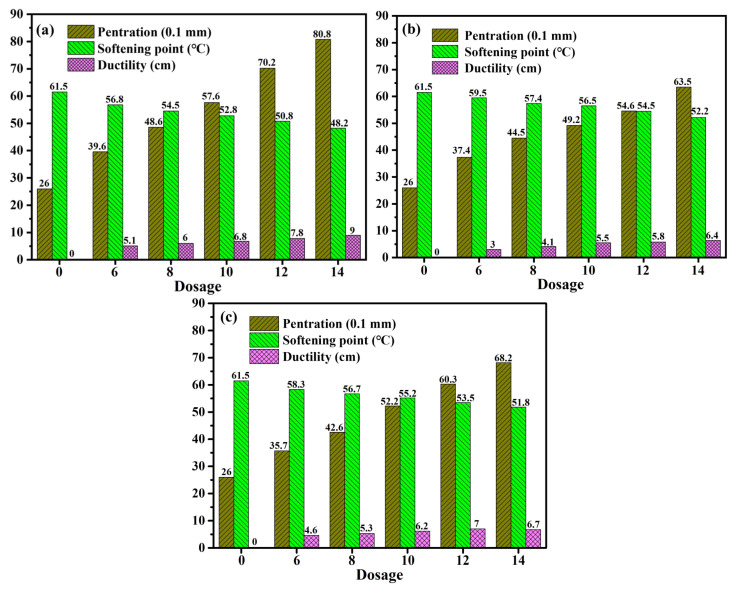
Three indicators of aged asphalt with different waste engine oil residues (WEORs): (**a**) WEOR-1; (**b**) WEOR-2; (**c**) WEOR-3.

Penetration can indicate the degree of softness and the relative viscosity of the asphalt binder. The softening point is an important indicator for evaluating the hardness and high-temperature performance of asphalt. Ductility can be used to evaluate the tensile deformation and flexibility of the asphalt binder. Figure 2 illustrates the three key indicators of aged asphalt subject to varying WEOR dosages (0%, 6%, 8%, 10%, 12%, and 14% aged asphalt qualities).

Recovery impacts of differing WEORs on the three primary indicators of aged asphalt reveal variations. As dosage escalates, the recovery effects on the three indicators augment but fail to revert to their original conditions. At a 14% WEOR-1 concentration, the recovery effect on the three major indicators of aged asphalt is optimal, though the recovery effect on ductility at 10 °C is subpar. Comparable findings apply to WEOR-2 and WEOR-3. Sun’s research [33] proposes that WEORs are unable to wholly reestablish the low-temperature performance of aged asphalt.

Given identical dosage, WEOR-1’s recovery effect on the three major indicators of aged asphalt surpasses those of WEOR-2 and WEOR-3. Nevertheless, the recovery rate of 10 °C ductility in aged asphalt from the three WEOR types is constrained, with a peak recovery of 36.9% from the original asphalt. Thus, this study advocates for the deployment of WEOR modification technology to offset the deficiencies in recovering aged asphalt performance.

### 3.2. Analysis of Dynamic Shear Rheometer (DSR) Rheological Performance Test Results

**Figure 3:**
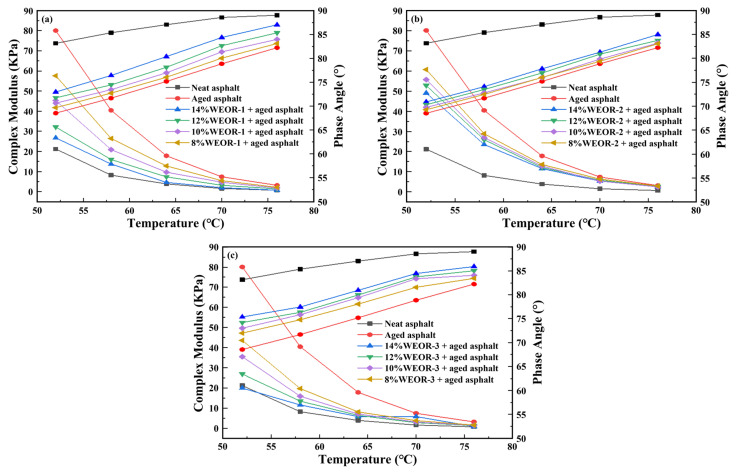
Test results of dynamic shear rheometer (DSR) with different WEOR dosages: (**a**) WEOR-1; (**b**) WEOR-2; (**c**) WEOR-3.

The DSR provided data, such as the phase angle (δ) and rutting factor (G*/sinδ), to assess the high-temperature rheological properties of asphalt mastics. Figure 3 displays results concerning the complex moduli and phase angles of aged asphalts under various WEOR dosages. Introducing a WEOR escalates the phase angle of aged asphalt while diminishing the complex modulus, suggesting a weakened deformation resistance in aged asphalt post-WEOR addition. It also implies a decline in high-temperature performance and a heightened risk of rutting. At a 14% dosage, WEOR-3 most effectively recovers the complex modulus of an aged asphalt, followed by WEOR-1 and WEOR-2. However, these fail to restore to the original asphalt level, implying the presence of unrecovered or less-recovered aged asphalt.

**Figure 4:**
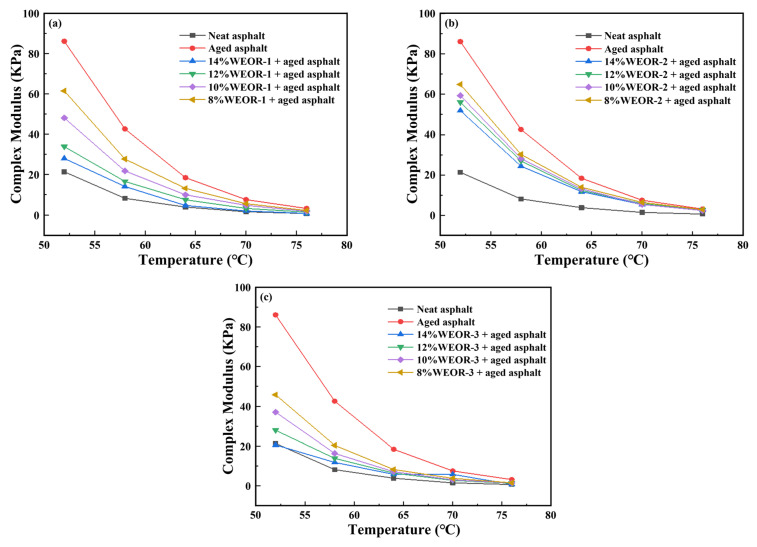
Anti-rutting Performance Curve of Aged Asphalt: (**a**) WEOR-1; (**b**) WEOR-2; (**c**) WEOR-3.

Figure 4 reveals that the aging asphalt exhibits the highest rutting resistance factor, while the matrix asphalt shows the lowest among all test samples. The asphalt’s high-temperature performance improves after aging. However, the addition of WEOR-1, WEOR-2, and WEOR-3 reduces the rutting resistance factor of aged asphalt, signifying that the high-temperature performance of aged asphalt diminishes with the inclusion of WEORs, thus elevating the risk of rutting. The greater the quantity of WEORs added, the more pronounced the decrease in the anti-rutting factor. When the content of WEOR-1 and WEOR-3 reaches 14%, the anti-rutting factor of WEORs modified asphalt sample approximates that of the original asphalt. Scholars such as Bennett [34] and Rubab [35] theorize that an excessive amount of WEOR may accelerate asphalt aging in subsequent stages, leading to a decline in low-temperature performance. In alignment with Ding’s [36] findings, this study recommends keeping the WEOR content below 14%.

### 3.3. Analysis of Four-Component Test Results in Thin-Layer Chromatography

A sample comprising 14% WEOR served as the representative for analyzing aged asphalt’s four-component alterations pre- and post-WEOR addition. Table 3 presents the thin-layer chromatography test results. The experimental data in the table led to the following observations.

The chemical compositions of WEORs, produced through varying treatment processes, display disparities, with WEOR-1 containing a higher percentage of saturate and aromatic components (89.4%)—a value substantially surpassing that of WEOR-2 (75.5%) and WEOR-3 (80.9%). This explains WEOR-1’s superior aged asphalt performance recovery effect relative to WEOR-2 and WEOR-3. Comparatively, the molecular distillation process outperforms the membrane treatment process in reclaiming waste engine oil’s light components. Conversely, post-distillation, WEOR-2 and WEOR-3 encompass significant molecular components such as asphaltene and resin.

WEOR can replenish the missing lightweight components in aged asphalt, thereby enhancing its performance. WEOR addition decreases aged asphalt’s heavy components, such as asphaltene, and augments light components, such as aromatic components. Nevertheless, none of the WEORs can reestablish the component proportions in aged asphalt to the level of original asphalt. For instance, with WEOR-1 addition, asphalt content in aged asphalt diminishes by 3.68%, resin content contracts by 4.29%, while aromatic content and saturate content increase by 6.76% and 1.21%, respectively. WEOR-2 and WEOR-3 follow identical increase and decrease trends.

In conclusion, while all three WEOR types enhance aged asphalt performance, the impact is relatively negligible, especially in terms of low-temperature performance recovery. Therefore, the direct application of WEORs for aged asphalt regeneration and utilization is not advised. This study, thus, amalgamated other modifiers to improve WEORs and develop better performing WEOR regeneration agents.

## 4. Development and Performance of WEOR Regenerant

### 4.1. Development of WEOR-G and WEOR-H Asphalt Recycling Agents

#### 4.1.1. Raw Materials and Technical Indicators

The investigation centered on WEOR-1 and WEOR-2, combining additives with various properties to develop a WEOR asphalt regeneration agent. Table 4 and Table 5 present the primary technical indicators of these additives.

For comparison and to assess the influence of the WEOR regenerative agent on the aged asphalt pavement performance, we selected the J-type regenerative agent. The manufacturer recommends a dosage constituting 10% of the aged asphalt’s quality. The J regeneration agent’s technical indicators can be found in Table 6.

#### 4.1.2. Research and Development of WEOR Asphalt Regeneration Agent

The WEOR-G asphalt regeneration agent contained five constituents: WEOR-1, liquid rubber, an ultraviolet absorber, a light shielding agent, and an antioxidant. Continuously stirred for 20–25 min using a JB300-SH medium- and low-speed mixer, the agent enhanced the regenerative effect of WEOR-1 on the aged asphalt. The proportions were as follows: 90.17% WEOR-1, 9.02% liquid rubber, 0.09% ultraviolet absorber, 0.45% light shielding agent, and 0.27% antioxidant. This composition implied an enhancement in thermal stability and resistance to low-temperature cracking.

The WEOR-H regenerative agent, composed of WEOR-2, aromatic oil, and liquid rubber, was compounded by stirring continuously for 20–25 min using the same mixer. The aromatic oil and liquid rubber constituted 8% and 6% of the WEOR-2 mass, respectively. By introducing these modifiers, the regenerative effect of WEOR-2 on the aged asphalt was bolstered.

### 4.2. Analysis of WEOR-G and WEOR-H Recycled Asphalt Test Results

#### 4.2.1. Three Major Indicators

**Figure 5:**
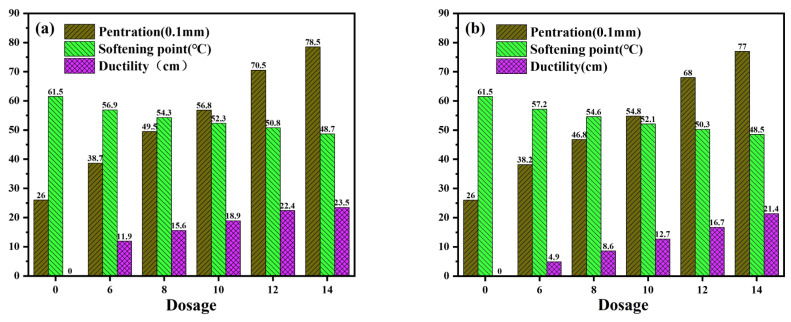
Three indicator results for aged asphalt: (**a**) WEOR-G; (**b**) WEOR-H.

The effects of WEOR-G, WEOR-H, and 10% J regenerants on three primary indicators of aged asphalt under varying dosages are presented in Figure 5 and Table 7. Some observations can be made:

The regenerative effects of the WEOR-G, WEOR-H, and J regenerants on aged asphalt outperform those of WEOR-1 and WEOR-2. Among these, WEOR-G displays the most effective regenerative properties, surpassing both the WEOR-H and J regenerative agents.

In comparison to WEOR-1 and WEOR-2, WEOR-G and WEOR-H significantly amplify the regenerative effects on aged asphalt. They can restore the three major indicators of aged asphalt to the original asphalt levels, specifically the 10 °C ductility. At a 14% dosage, WEOR-G and WEOR-H can elevate them to 23.5 and 21.4 cm, respectively, effectively offsetting the limited capacity of WEOR-1 and WEOR-2 to rehabilitate the low-temperature performance of aged asphalt. The primary reason for this lies in the addition of liquid rubber components to WEOR-G and WEOR-H. These components interact with the aged asphalt, leading to swelling and slight cross-linking reactions. By absorbing the lightweight components in the asphalt, they enable swelling, resulting in cohesive forces and enhancing the low-temperature performance.

#### 4.2.2. High-Temperature Rheological Performance

**Figure 6:**
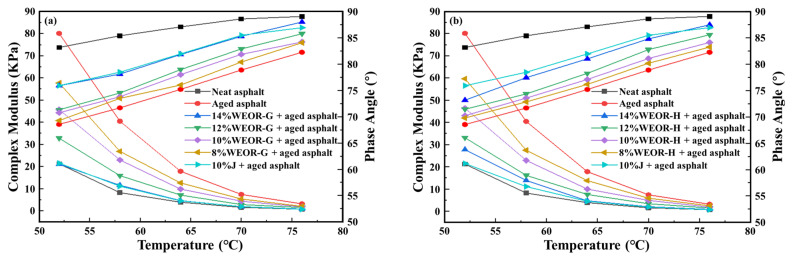
Test results for rheological properties of aged asphalt: (**a**) WEOR-G; (**b**) WEOR-H.

Figure 6 exhibits the experimental results for the complex modulus and phase angle of aged asphalt with different dosages of WEOR-G, WEOR-H, and the 10% J regenerant. Considering the data in Figure 3, the introduction of WEOR-G, WEOR-H, and J-type regenerants enhances the phase angle of aged asphalt and diminishes its complex modulus, suggesting a decline in high-temperature performance of aged asphalt post-addition of these regenerants. As the concentration of the three regenerants increases, the complex modulus and phase angle of aged asphalt gradually approximate those of the original asphalt. At a 14% dosage of WEOR-G and WEOR-H, the complex modulus of the aged asphalt essentially recovers to the original asphalt level. The regenerative effect is marginally superior to that of the J regenerant and considerably more potent than those of WEOR-1 and WEOR-2.

**Figure 7:**
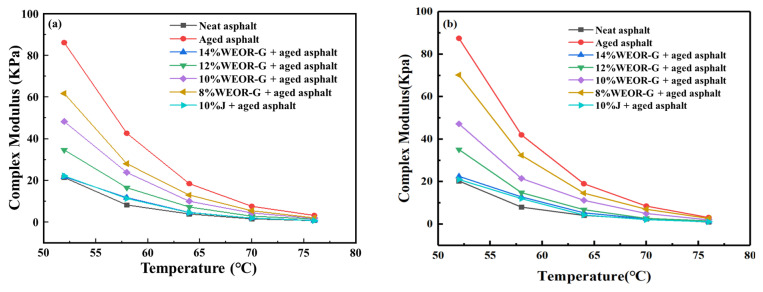
Anti-rutting Performance Curve of Aged Asphalt: (**a**) WEOR-G; (**b**) WEOR-H.

From the experimental data in Figure 7, it is evident that the addition of WEOR-G, WEOR-H, and J-type recycling agents decreases the anti-rutting factor of aged asphalt. This indicates a reduction in the high-temperature performance of aged asphalt after the inclusion of these three types of recycling agents. When the dosage of WEOR-G reaches 14%, the anti-rutting factor of the WEORs modified asphalt sample essentially reduces to the level of the original asphalt, and its performance is equivalent to that of the J regeneration agent at a 10% dosage. Consequently, this study recommends limiting the content of WEOR-G and WEOR-H to 14% to ensure the continued performance of the aged asphalt. The paper selects 14% WEOR-G and WEOR-H recycled asphalt as representative, comparing the low-temperature performance of recycled asphalt with J-type regenerant.

#### 4.2.3. Analysis of Low-Temperature Performance Test Results

**Figure 8:**
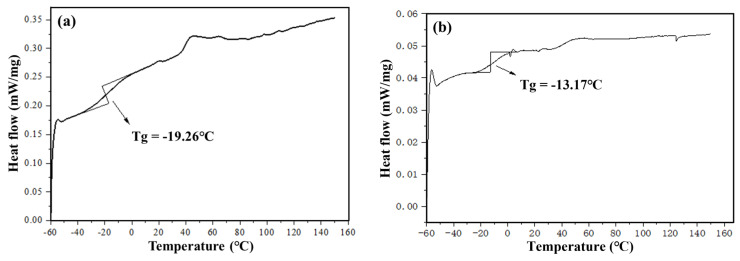

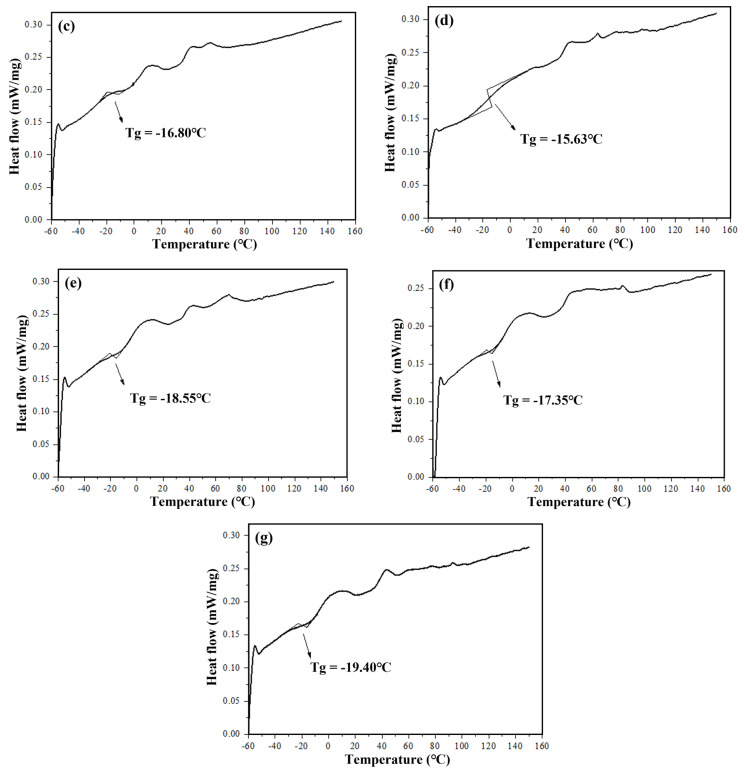
Differential scanning calorimetry curves and glass transition temperatures of asphalts: (**a**) original asphalt; (**b**) aged asphalt; (**c**) 14% WEOR-1 + aged asphalt; (**d**) 14% WEOR-2 + aged asphalt; (**e**) 10% J + aged asphalt; (**f**) 14% WEOR-H recycled asphalt; (**g**) 14% WEOR-G recycled asphalt.

The heat flow curve of the asphalt sample within the temperature range of −60 °C to 160 °C, as seen in Figure 8, reveals a reduction in the glass transition temperature of aged asphalt following the addition of WEOR-1, WEOR-2, WEOR-H, WEOR-G, and J regenerants. The glass transition temperature decreases in the following order: aged asphalt (−13.17 °C) > WEOR-2 asphalt (−15.63 °C) > WEOR-1 asphalt (−16.80 °C) > WEOR-H recycled asphalt (−17.35 °C) > J recycled asphalt (−18.55 °C) > original asphalt (−19.26 °C) > WEOR-G recycled asphalt (−19.40 °C). Compared to WEOR-1 and WEOR-2, both WEOR-H and WEOR-G can lower the glass transition temperature of recycled asphalt. The primary reason for this is that liquid rubber decreases the temperature sensitivity of the aged asphalt, endowing it with superior low-temperature deformation capabilities and boosting its resistance to low-temperature cracking.

## 5. Discussion

According to the results of previous studies, the inclusion of WEOR to aged asphalt can lead to the performance restoration to some extent [37]. The light components in WEOR contribute to the dissolution of asphaltenes, thus softening the aged binder [38,39]. However, adding WEOR to neat asphalt binder lowers the elastic recovery ability and deformation resistance at high temperatures, leading to a decreased rutting resistance of asphalt [30]. Wang’s results show that there is no decrease in high-temperature performance for the aged asphalt modified by styrene-butadiene-styrene (SBS), after regeneration by WEOR. The reason is that the light components induce the swelling of SBS network, restricting the thermal motion of asphalt molecules. Unfortunately, the viscous property of asphalt has not been restored due to the presence of SBS [39]. The results of this study also proved that there are both positive and negative effects on the performance of asphalt binder regenerated by WEOR. Excessive additives should be introduced to the WEOR to obtain a high-performance asphalt regenerant. More research needs to be conducted to ascertain the effects of the excessive additives on the performance of WEOR-regenerated asphalt in future.

Most of the chemical compounds in WEOR are light components with molecular weights of less than 200 g/mol, for instance, paraffin oil, polyolefin oil, aromatic solvents, and so on. These are similar to the light components of asphalt binder, allowing WEOR to be compatible with asphalt easily [40]. However, the light components from WEOR, easily volatile, lead to a decrease in the secondary aging resistance of WEOR-rejuvenated asphalt [10]. That is to say, the long-term aging resistance property of WEOR-rejuvenated asphalt binder is poor. Furthermore, WEOR has relatively higher levels of heavy metals such as Fe, Cd, Cr, Pb, etc., [41]. These metals can cause pollution of the groundwater and soil when discharged into land or a water source, so are thereby harmful to human health. As such, it is necessary to perform pre-treatment on WEOR before using it as asphalt regenerant to ensure its purity and avoid environmental pollution. 

## 6. Conclusions

This investigation has yielded several key findings:

- The use of WEOR can modify the proportion of four principal components in aged asphalt. Specifically, it can elevate lighter constituents, such as aromatic components, while reducing heavier ones, such as asphaltene. This alteration can improve the three major indicators of aged asphalt. However, the regenerative effect of WEOR is limited, particularly in terms of low-temperature performance. As a result, it is not advisable to use WEOR solely as an asphalt regeneration agent.
- WEOR can enhance the phase angle of aged asphalt while decreasing its complex modulus. This implies that WEOR can lead to a decline in the high-temperature performance of aged asphalt, thereby increasing the likelihood of rutting. Accordingly, the study recommends limiting the WEOR dosage to less than 14%.
- WEOR-G asphalt regeneration agent is formulated using WEOR-1, liquid rubber, ultraviolet absorber, light shielding agent, and antioxidant. WEOR-H asphalt regeneration agent is created through WEOR-2, aromatic oil, and liquid rubber. The findings show that WEOR-G and WEOR-H notably enhance the regenerative effect of aged asphalt, and can increase the 10 °C ductility to 23.5 and 21.4 cm (Original asphalt is 24.4 cm), effectively compensating for the limited ability of WEOR-1 and WEOR-2 to restore the low-temperature performance of aged asphalt.
- The study discerned a hierarchy in the regenerative effect of each material on aged asphalt. WEOR-G, the J-type regeneration agent, and WEOR-H were ranked in descending order of effect, with WEOR-G demonstrating the most potent regenerative effect. This substance also exhibited the lowest glass transition temperature and superior resistance to low-temperature deformation.
- When the dosage of WEOR-G is 14%, the WEOR recycled asphalt sample exhibits strong low-temperature performance, and its anti-rutting factor is fundamentally equivalent to that of the original asphalt. WEOR-G has a pronounced effect on the regeneration of aged asphalt. Therefore, this study recommends using WEOR-G as an asphalt regeneration agent to enhance the road performance of aged asphalt.

## Figures and Tables

**Table 1:** Basic physical indicators of waste engine oil residues (WEORs).

Index	Unit	WEOR-1	WEOR-2	WEOR-3
Exterior	—	grey green	dark green	black
Density (15 °C)	g/cm^3^	0.9370	0.9875	1.010
Kinematic viscosity (40 °C)	mm^2^/s	74.21	125.77	553.72
Flash point (opening)	°C	198	204	230
Mechanical impurities	%	0.094	0.133	0.157
Ash content	%	0.033	3.851	4.710
Quality changes before and after RTFOT	%	3.833	1.695	0.462

**Table 2:** Test results of 70# base asphalt before and after aging.

Aging Degree	Aging Time (min)	Technical Indicators	Unit	Shell 70#
Original asphalt	0	Penetration (25 °C, 5 s, 100 g)	0.1 mm	75
		Softening point	°C	48.5
		10 °C ductility (5 cm/s)	cm	24.4
Aged asphalt	270	Penetration (25 °C, 5 s, 100 g)	0.1 mm	26
		Softening point	°C	61.5
		10 °C ductility (5 cm/s)	cm	0

**Table 3:** Basic physical indicators of WEORs.

Type	Saturate (%)	Aromatic (%)	Resin (%)	Asphaltene (%)
WEOR-1	18.1	71.3	8.5	2.1
WEOR-2	25.7	49.8	16.0	8.5
WEOR-3	22.8	58.1	14.2	4.9
Original asphalt	23.91	43.17	23.13	11.79
Aged asphalt	19.14	30.47	33.00	17.39
14% WEOR-1 asphalt sample	20.35	37.23	28.71	13.71
14% WEOR-2 asphalt sample	19.51	33.28	30.83	16.38
14% WEOR-3 asphalt sample	19.81	35.78	29.28	15.13

**Table 4:** Technical indicators of aromatic oil.

Color	Aromatic Content (%)	Ash Content (%)	Flash Point (°C)	15 °C Density (g/cm^3^)	Kinematic Viscosity (100 °C, mm/s)
blackish green	84.5	0.05	236	1.018	28

**Table 5:** Technical indicators of liquid rubber.

State	Molecular Weight	Ash Content (%)	Styrene Content (%)	Tensile Strength (MPa)	Mooney Viscosity (100 °C)	Elongation at Break (%)
White viscous	50,000	0.05	26.5	19	56	609

**Table 6:** Indicators of J regeneration agent.

Type	Viscosity (60 °C, Pa·s)	Flash Point (°C)	Saturation Content (%)	Aromatic Content (%)	Viscosity Change before and after RTFOF	Quality Change before and after RTFOF (%)
Regenerant J	5268	242	20.5	63.8	1.2	0.6

**Table 7:** Test results for three indicators of J recycled asphalt.

Asphalt Type	Penetration (0.1 mm)	Softening Point (°C)	Ductility (mm)
Recycled asphalt J	81	47.3	21.1

## Data Availability

No new database has been created, please contact the author if required.
